# Person-to-Person Transmission of Andes Virus (ANDV): A Systematic Review of Transmission Dynamics, Viral Shedding, and Public Health Implications

**DOI:** 10.3390/v18070699

**Published:** 2026-06-25

**Authors:** Flavia Pennisi, Antonio Pinto, Stefania Borlini, Sabrina Caruccio, Giusy D’Alterio, Carlo Signorelli, Giovanni Rezza

**Affiliations:** 1 Faculty of Medicine, Vita-Salute San Raffaele University, 20132 Milan, Italy; pennisi.flavia@hsr.it (F.P.); caruccio.sabrina@hsr.it (S.C.); dalterio.giusy@hsr.it (G.D.); signorelli.carlo@hsr.it (C.S.); rezza.giovanni@hsr.it (G.R.); 2 PhD National Program in One Health Approaches to Infectious Diseases and Life Science Research Department of Public Health, Experimental and Forensic Medicine, University of Pavia, 27100 Pavia, Italy; 3 ASST Papa Giovanni XXIII, 24127 Bergamo, Italy; sborlini@asst-pg23.it

**Keywords:** Andes virus, Andes orthohantavirus, hantavirus cardiopulmonary syndrome, person-to-person transmission, viral shedding, viral load, incubation period, serial interval, contact tracing, outbreak preparedness

## Abstract

Andes virus (ANDV) is the only hantavirus with well-documented evidence of person-to-person transmission. However, key parameters related to transmission timing, viral shedding, exposure contexts, and public health management remain incompletely defined. We conducted a systematic review in accordance with PRISMA 2020. MEDLINE/PubMed, Scopus, and Web of Science were searched from database inception up to 14 May 2026. Eligible studies reported epidemiological, virological, clinical, or public health data relevant to ANDV infection, person-to-person transmission, viral shedding, and/or outbreak control. Thirty-three studies, including 17,204 individuals, 2221 laboratory-confirmed ANDV cases, and 135 documented secondary cases, were included. Person-to-person transmission was identified as a primary or co-occurring route in 20 papers. The median incubation period among ANDV cases was 20.8 days, and the median serial interval was 21.8 days (upper bounds near 40 days). Secondary attack rates were higher among sexual and other close contacts. ANDV RNA was consistently detected in blood and occasionally in saliva, respiratory secretions, urine, breast milk, and semen, although RNA detection alone does not necessarily imply infectious virus. Rare reports of culture-confirmed isolation of replication-competent virus support the biological plausibility of transmission via close mucosal or respiratory exposure. Unlike other hantaviruses, Andes virus can spread person to person through close contact, supporting prolonged monitoring and risk-stratified follow-up of high-risk contacts based on ANDV-specific epidemiological evidence. Possible recommendations, including post-discharge counselling regarding possible sexual transmission, remain provisional and require further evidence. Preparedness activities against outbreaks should also be implemented in non-endemic regions, while future research should prioritize prospective contact studies, standardized virological sampling, and genomic confirmation.

## 1. Introduction

Hantaviruses (family Hantaviridae, genus Orthohantavirus) are rodent-borne zoonotic pathogens that cause two major clinical syndromes in humans: hemorrhagic fever with renal syndrome (HFRS), which occurs mainly in Europe and Asia, and hantavirus cardiopulmonary syndrome (HCPS), which occurs in the Americas and is characterized by acute respiratory failure, cardiogenic shock, and case-fatality ratios often exceeding 30% [1]. The primary route of human infection for virtually all hantaviruses is the inhalation of aerosolized rodent excreta, including urine, feces, and saliva, from chronically infected reservoir hosts, without evidence of direct human-to-human transmission [2].

Andes orthohantavirus (ANDV; Orthohantavirus andesense), first characterized in Argentina in 1995–1996, is a leading cause of HCPS in the Southern Cone of South America and is responsible for a large proportion of reported cases in Argentina and Chile [3]. Its principal reservoir is the long-tailed pygmy rice rat (Oligoryzomys longicaudatus), a rodent reservoir host distributed in southern Argentina and Chile [4].

What distinguishes ANDV from other hantaviruses is its documented capacity for person-to-person transmission: an outbreak identified in southwestern Argentina in 1996 provided the first clear evidence of interhuman spread and established ANDV as the only hantavirus with well-documented sustained person-to-person transmission [5]. Since then, additional transmission events have been documented in Argentina and Chile through epidemiologically and molecularly linked cluster investigations in household, nosocomial, social, and travel-related settings [6,7]. More recently, Andes virus was identified as the cause of a cluster of illness on a cruise ship, further highlighting its international public health relevance [8].

Despite this distinctive capacity for person-to-person transmission, critical parameters governing the dynamics of interhuman spread remain incompletely characterized. In particular, the timing of infectiousness relative to clinical onset, including the possibility of pre-symptomatic or early symptomatic transmission, has not yet been systematically characterized, although cluster investigations suggest that transmission may occur during the prodromal phase or shortly thereafter [9,10]. In observational investigations of documented human-source exposure, the incubation period of ANDV-associated HCPS has been estimated to range between 7 and 39 days, with a median of approximately 18 days, complicating contact tracing and follow-up strategies [11].

Recent prospective evidence has shown that ANDV RNA can be detected in multiple biological specimens during acute infection, including blood, saliva, and respiratory secretions, helping to clarify the kinetics of viraemia and viral shedding in humans [12]. Experimental and ultrastructural findings also support respiratory and/or salivary pathways as plausible mechanisms of interhuman transmission [13]. In addition, a new ANDV isolate haplotype was recently obtained during prospective follow-up of close contacts of a fatal ANDV-associated HCPS case in Chile, providing further virological evidence consistent with ongoing human-to-human transmission [14].

The biological basis of contagiousness nevertheless remains incompletely defined. Available studies on the duration and magnitude of viraemia and on shedding across different biological matrices are still limited, although ANDV RNA has been documented in semen [15]. Likewise, seroprevalence data suggest that asymptomatic infection nay occur, but its frequency and epidemiological significance have not been systematically established [16].

The contexts and determinants of secondary transmission are heterogeneous and incompletely described. Prospective follow-up of household contacts in Chile identified secondary infections associated with close physical contact during the symptomatic period, whereas the 2018–2019 Chubut outbreak showed that, under specific epidemiological conditions, ANDV can sustain transmission chains beyond the household setting, including super-spreading events, with a reproductive number estimated above 2.0 before control measures were implemented [6,7]. Household and nosocomial transmission described in Chile further emphasized the role of healthcare settings as potential amplification environments and the need for stringent infection prevention measures and clear definitions of high-risk contacts [17]. To our knowledge, no systematic review has specifically synthesized the epidemiological, virological, and public health evidence on person-to-person transmission of ANDV. Given the growing concern over emerging high-consequence viral pathogens, and considering ANDV’s unique status among hantaviruses, a rigorous evidence synthesis is needed to inform infection prevention practices, contact tracing protocols, and outbreak preparedness planning [18].

This systematic review, therefore, aims to synthesize the available evidence on person-to-person transmission of Andes virus, with specific focus on the following: (i) the timing of infectiousness relative to symptom onset, including possible pre-symptomatic transmission; (ii) the occurrence of asymptomatic and paucisymptomatic ANDV infections; (iii) the detection of viraemia, viral load, and viral shedding across biological specimens; (iv) exposure contexts and factors associated with secondary transmission; and (v) public health measures adopted in documented outbreaks, including contact tracing, isolation, quarantine, and definitions of high-risk contacts.

## 2. Materials and Methods

### 2.1. Study Design and Protocol Registration

This systematic review was conducted in accordance with the Preferred Reporting Items for Systematic Reviews and Meta-Analyses (PRISMA 2020) statement [19], Supplementary Table S2. The review protocol was developed a priori and prospectively registered in the International Prospective Register of Systematic Reviews (PROSPERO; registration number: CRD420261397971). The review was designed to systematically summarize the available evidence on person-to-person transmission of Andes virus (ANDV), with a specific focus on transmission dynamics, viral shedding, timing of infectiousness, asymptomatic or paucisymptomatic infections, exposure contexts, and public health measures adopted during documented outbreaks or transmission events.

### 2.2. Terminology

Throughout this review, “ANDV infection” refers to confirmed, probable, or suspected infection with Andes virus, including symptomatic, paucisymptomatic, and asymptomatic infections, as defined by the original study authors. “ANDV-associated HCPS” refers specifically to the clinical syndrome caused by ANDV infection. Because the terms hantavirus pulmonary syndrome (HPS) and hantavirus cardiopulmonary syndrome (HCPS) are both used in the source literature, we use HCPS as the preferred term in this manuscript, while retaining HPS only when reflecting terminology used in the original studies, search strategies, or tables. The broader expression “ANDV-associated disease” was avoided where possible and replaced with either “ANDV infection” or “ANDV-associated HCPS”, depending on the context.

### 2.3. Review Question and Eligibility Criteria

The review question and eligibility criteria were defined according to the PICOS framework:

- Population (P): Humans with confirmed, probable, or suspected Andes virus (ANDV) infection, as defined by the original study authors, and their household, healthcare, or community contacts. Studies involving the natural reservoir, particularly Oligoryzomys longicaudatus, were considered eligible when relevant to the ecological or epidemiological context of ANDV transmission.
- Exposure (I/E): Exposure to an ANDV-infected individual in a person-to-person transmission context, environmental or animal exposure to ANDV when relevant for distinguishing alternative transmission routes, or biological specimens collected from infected individuals for the assessment of viraemia, viral load, or viral shedding.
- Comparator (C): No comparator was required for inclusion. When reported, eligible comparators included uninfected contacts, asymptomatic versus symptomatic infections, or different exposure settings.
- Outcomes (O): The primary outcome was documented or probable person-to-person transmission of ANDV, including transmission events and transmission chains. Secondary outcomes included timing of transmission in relation to symptom onset, viraemia and viral shedding across biological matrices, incubation period, serial interval, secondary attack rate, asymptomatic or paucisymptomatic infection, and public health measures, such as contact tracing, isolation, quarantine, and contact-risk definitions.
- Study design and setting (S): Eligible studies included outbreak investigations, case reports, case series, cohort studies, case–control studies, cross-sectional studies, seroprevalence or serosurvey studies, virological studies, and ecological or geographical studies. Public health documents, guidelines, rapid risk assessments, and technical reports from institutional bodies were also considered when reporting data relevant to contact management, isolation, quarantine, contact tracing, or risk assessment. Studies were eligible if they reported data on ANDV, Andes orthohantavirus, Orthohantavirus andesense, or ANDV-associated HCPS in epidemiological contexts compatible with ANDV circulation or in settings where Oligoryzomys longicaudatus was identified as the relevant reservoir.

Studies were excluded if they (i) focused exclusively on hantaviruses other than ANDV and data were not separable; (ii) reported generic hantavirus data without specific attribution to ANDV infection, ANDV-associated HCPS, or a clearly compatible ANDV epidemiological context; (iii) were exclusively experimental “in vitro” or animal studies, without relevance to human infection, transmission, viral shedding, natural reservoir ecology, or public health management; or (iv) were diagnostic or methodological studies not reporting transmission, temporal specimen positivity, viral load, viraemia, clinical cases, or control measures. Reviews, editorials, commentaries, and letters without original data were excluded from primary data extraction but screened for additional references. No restrictions were applied by publication year; however, only studies published in English were included.

### 2.4. Information Sources and Search Strategy

A comprehensive literature search was conducted in MEDLINE via PubMed, Scopus, and Web of Science from database inception to 14 May 2026. The primary search strategy for PubMed was constructed by combining MeSH terms and free-text keywords related to the following: (i) Andes virus and ANDV-associated HCPS, including related taxonomic and clinical terms; (ii) person-to-person transmission, transmission chains, outbreak investigations, and exposure contexts; and (iii) viral shedding, viraemia, viral load, biological specimens, and public health measures, including contact tracing, isolation, and quarantine. Search strings were adapted to the syntax and indexing structure of each database. The full search strategy for each database is reported in Supplementary Table S1.

Additional relevant records were identified through grey literature and institutional website searches, as well as backward and forward citation tracking of included studies and key articles.

### 2.5. Study Selection

All retrieved records were imported into Rayyan (Rayyan Systems Inc., Cambridge, MA, USA), and duplicates were removed. Three reviewers independently screened titles and abstracts, followed by full-text assessment of potentially eligible studies. Any uncertainty or disagreement was resolved through discussion and, when needed, by consultation with a fourth reviewer. Reasons for exclusion at the full-text stage were recorded in a dedicated screening file.

### 2.6. Data Extraction

Data were extracted independently by four reviewers using a standardized Microsoft Excel form developed for this review and organized into study-level, transmission, virological, and risk-of-bias domains. Discrepancies were resolved by consensus and, when required, by consultation with another member of the review team. When multiple reports referred to the same outbreak, cluster, or transmission chain, data were cross-checked across sources and consolidated to avoid double counting. Overlap was assessed by comparing country, region, study period, outbreak dates, case numbers, transmission setting, and reported epidemiological links. When surveillance datasets and outbreak-specific reports overlapped, denominators were extracted conservatively: study-level totals were included only once, and more detailed outbreak reports were used to characterize transmission dynamics without adding duplicate individuals or cases to the overall counts.

For each included study, the following variables were extracted when available:

- Study characteristics: first author, year of publication, DOI, country, WHO region, income level, study design, data source, study period, setting, number of total cases, number of confirmed ANDV cases, number of secondary cases, diagnostic confirmation method, and reservoir information.
- Transmission-related data: type and context of transmission, evidence supporting person-to-person spread, number of transmission pairs, timing of exposure in relation to symptom onset, pre-symptomatic transmission, asymptomatic or paucisymptomatic infection, incubation period, serial interval, secondary attack rate, household or healthcare-associated transmission, healthcare worker involvement, personal protective equipment use, and evidence supporting or excluding shared environmental or rodent exposure.
- Public health measures: contact tracing, contact definitions, risk stratification, isolation, quarantine duration, follow-up period, and other outbreak-control measures.
- Virological data: specimen type, timing of collection, days from symptom onset, laboratory method, viral RNA detection, Ct values or viral copy number, infectious virus isolation, viraemia, specimen-specific positivity, serial sampling, shedding duration, and timing of viral-load peak.
- Risk-of-bias information: JBI tool applied, domain-level judgements, outbreak-specific items, virological supplementary criteria, overall risk-of-bias judgement, and reviewer notes.

When information was incomplete or inconsistently reported, data were extracted conservatively from the published text, tables, figures, or supplementary materials. For overlapping estimates, priority was given to laboratory-confirmed ANDV cases, explicitly documented transmission links, and reports providing the clearest temporal reconstruction.

### 2.7. Data Synthesis and Statistical Analysis

Given the clinical and methodological heterogeneity of the included studies, a formal meta-analysis was not performed. Data were synthesized narratively in accordance with the Synthesis Without Meta-analysis (SWiM) reporting guideline. For quantitative epidemiological parameters, numerical estimates were extracted from eligible studies and summarized descriptively as medians, means, standard deviations, interquartile ranges, and overall observed ranges. For studies reporting ranges without a central estimate, midpoint values were used for descriptive purposes and clearly distinguished from directly reported point estimates. Estimates derived from confirmed person-to-person transmission events were analyzed separately from those derived from suspected or mixed-exposure contexts. All numerical summaries were considered exploratory descriptive summaries and were not interpreted as pooled effect estimates. Midpoint-derived values were used only to describe the distribution of reported data when no central estimate was available, and no inferential weighting or meta-analytic pooling was applied.

For interpretative purposes, transmission evidence was stratified according to certainty of attribution: (i) genomically confirmed person-to-person transmission, when epidemiologically linked cases were supported by viral sequencing, phylogenetic clustering, or closely related viral genomes; (ii) epidemiologically documented person-to-person transmission, when close-contact exposure, compatible temporality, and no plausible shared rodent or environmental exposure supported interhuman spread in the absence of genomic confirmation; (iii) suspected/probable person-to-person transmission, when close-contact exposure and timing were compatible with interhuman transmission but contact reconstruction was incomplete or shared exposure could not be fully excluded; and (iv) mixed/uncertain exposure, when both person-to-person and zoonotic/environmental exposure remained plausible and attribution to a single route was not possible. In the narrative synthesis, findings were interpreted according to the directness and certainty of the underlying evidence. Greater weight was assigned to outbreak investigations, prospective contact studies, and virological studies with laboratory confirmation, compatible temporality, genomic support, or exclusion of shared environmental exposure. Case reports, serosurveys, ecological studies, and public health documents were used mainly as contextual or supportive evidence and were not considered sufficient, in isolation, to establish transmission parameters or causal person-to-person transmission links.

Virological data were synthesized descriptively, with proportions calculated across studies reporting each outcome. Given the high proportion of studies that did not assess specific biological compartments, observed positivity proportions are reported as fractions of studies that explicitly tested each specimen type, rather than as population-level estimates. Culture-confirmed isolation of replication-competent virus was analyzed separately from RNA-only detection data. Molecular detection of ANDV RNA was, therefore, considered evidence of viral genetic material in the tested specimen, whereas culture-confirmed isolation of replication-competent virus was considered stronger evidence of potential infectivity. Narrative subgroup syntheses were constructed for the following: (i) blood and plasma viraemia; (ii) extravascular shedding compartments (saliva, urine, respiratory secretions, semen); and (iii) longitudinal shedding kinetics from studies performing serial sampling.

### 2.8. Risk of Bias and Methodological Quality Assessment

Risk of bias and methodological quality were assessed independently by four reviewers using Joanna Briggs Institute critical appraisal tools [20,21] selected according to study design. Case reports, case series, cohort studies, case–control studies, cross-sectional studies, and prevalence studies were assessed using the corresponding JBI checklists. Disagreements were resolved by discussion or, when necessary, by consultation with another reviewer.

Because a substantial proportion of the evidence was expected to derive from outbreak investigations and descriptive public health reports, standard appraisal was supplemented with outbreak-specific criteria. Virological studies were additionally assessed for precision in reporting specimen collection timing relative to symptom onset, specimen type, laboratory method validation, use of serial sampling, and distinction between viral RNA detection and infectious virus isolation.

Each study was assigned an overall methodological judgement based on the relevant checklist and supplementary criteria, taking into account the study design, completeness of reporting, internal consistency, and relevance to the review question.

### 2.9. Ethics

As this study was based exclusively on previously published or publicly available data, ethics approval and informed consent were not required.

## 3. Results

### 3.1. Literature Search

**Figure 1:**
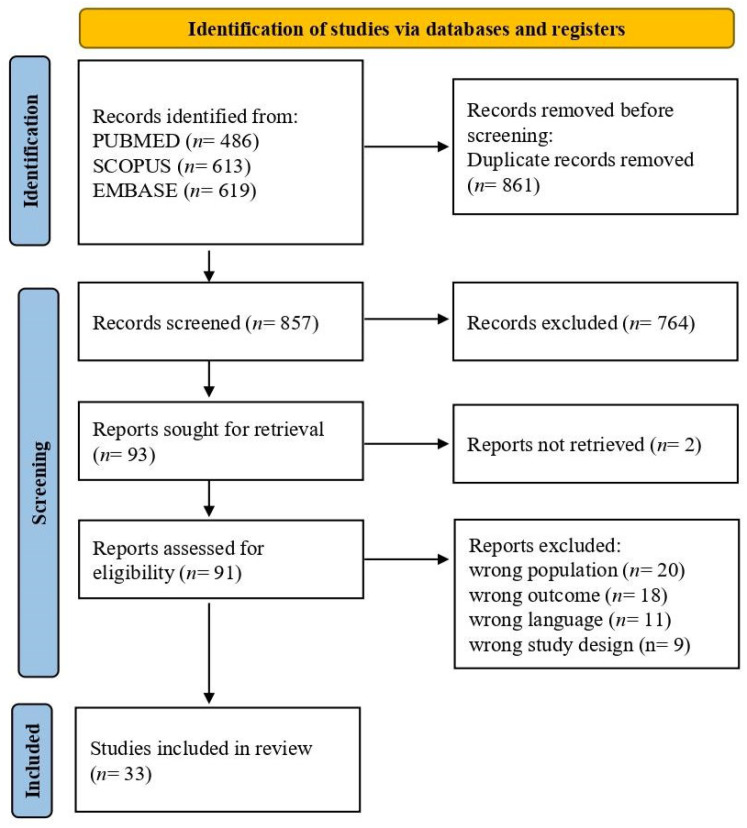
PRISMA 2020 flow diagram of the study selection process for the systematic review.

The literature search yielded 1718 records across PubMed/MEDLINE (*n* = 486), Scopus (*n* = 613), and Web of Science (*n* = 619). After duplicate removal, 857 records were screened by title and abstract, of which 764 were excluded. Full-text retrieval was attempted for 93 reports; 2 could not be obtained, and 91 reports were assessed for eligibility. Of these, 58 were excluded because of wrong population (*n* = 20), wrong outcome (*n* = 18), wrong language (*n* = 11), or wrong study design (*n* = 9). Therefore, 33 studies met the inclusion criteria and were included in the review [3,6,7,9,10,11,12,13,14,15,17,22,23,24,25,26,27,28,29,30,31,32,33,34,35,36,37,38,39,40,41,42,43]. The selection process is presented in the PRISMA 2020 flow diagram (Figure 1).

### 3.2. Geographical Distribution

**Figure 2:**
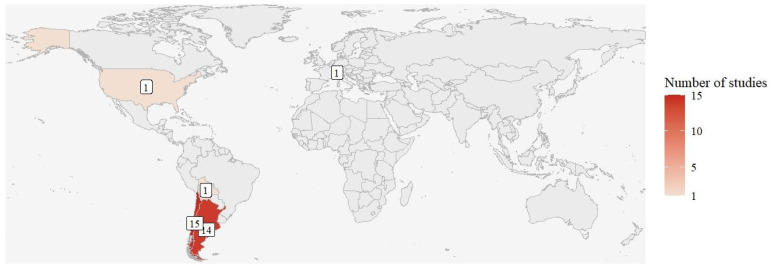
Geographical distribution of included studies by country. Only studies attributable to a single-country setting were mapped; one multinational study was excluded from the geographical display.

The geographical distribution of the included studies is presented in Figure 2. A global projection was intentionally retained to visually highlight the marked concentration of evidence in the Southern Cone and the absence of included studies from other world regions. Of the 33 studies included in this review, 32 were attributable to a single-country setting and were, therefore, represented on the map, whereas 1 multinational study was not displayed separately. The available evidence was highly concentrated in South America, particularly in Chile (*n* = 15) [6,11,12,13,14,17,28,29,31,32,36,37,39,41,42] and Argentina (*n* = 14) [7,9,10,22,23,24,25,26,27,30,33,35,38,43], which together accounted for the vast majority of country-specific studies. Only a limited number of studies originated from other settings, with one study each from Bolivia [40], the United States of America (USA) [34], and Switzerland [15]. Instead, one study had a multinational setting: Padula et al. pooled hantavirus/ANDV-related clinical, serological, and genetic data from collaborating centers across five South American countries and was, therefore, not attributable to a single country for mapping purposes [3]. Overall, the geographical pattern highlights a marked concentration of the literature in the Southern Cone, with minimal representation from other regions. More detailed information on the specific regions, provinces, cities, or exposure locations reported in each study is provided in Supplementary Table S3.

### 3.3. Study Characteristics

The 33 included studies encompassed a total of 17,204 individuals across all study populations, of whom 2221 were laboratory-confirmed ANDV cases in the 29 studies [6,7,9,10,11,12,13,14,15,17,22,24,25,26,28,29,30,31,32,33,34,35,36,37,38,39,40,41,42] providing this information, and 135 documented secondary cases in the 15 studies [6,7,9,10,12,14,17,24,25,28,31,33,34,35,42] reporting this outcome. Studies were published between 1997 [27,43] and 2025 [14,24]. A notable increase in scientific output was observed after 2019: 10 studies (27.3%) [7,10,12,14,15,23,24,31,33,37] were published between 2020 and 2025, reflecting renewed attention following the Chubut 2018–2019 outbreak [7] and emerging virological evidence from Chile. The full characteristics of all included studies are presented in Table 1.

In terms of study design, outbreak investigations were the most frequently represented type (*n* = 9) [7,9,10,17,27,34,35,42,43], followed by virological studies (*n* = 8) [13,14,15,22,23,24,25,30], case series (*n* = 6) [3,11,29,37,38,41], prospective cohort studies (*n* = 4) [6,12,32,33], seroprevalence or serosurvey studies (*n* = 2) [28,39], case reports (n = 2) [31,40], retrospective cohort studies [36], and ecological studies [26] (*n* = 1 each).

The primary reservoir was found to be Oligoryzomys longicaudatus in 12 studies [6,11,22,24,27,28,32,33,34,36,38,40]; 5 studies reported other rodent species [7,10,12,29,37,43].

As shown in Table 2, ANDV infection was confirmed in all studies except three studies that reported HCPS without specific ANDV serotype attribution [3,26,27]. The main diagnostic method was PCR combined with serology, performed in all studies except five where serology alone was performed [13,27,28,29,41] and one study where PCR alone was performed [23].

### 3.4. Person-to-Person Transmission

#### 3.4.1. Transmission Classification and Contexts

As shown in Table 2, evidence compatible with P2P transmission was identified in 20 of 33 included studies [3,6,7,9,10,12,13,14,17,22,23,27,28,31,32,33,35,38,42,43]. These studies were not treated as a single evidentiary category. Instead, they were separated according to certainty of attribution: documented P2P transmission without a clearly co-occurring zoonotic exposure was reported in 5 studies [10,31,35,38,40], suspected/probable P2P transmission in 11 studies [12,13,14,17,22,27,28,32,38,42,43], and mixed or co-occurring P2P and possible zoonotic/environmental exposure in 4 studies [6,9,23,33]. The remaining studies reported either exclusively zoonotic exposure or did not report the transmission route. Among P2P-related studies, documented transmission was less frequent than suspected transmission, reflecting the difficulty of excluding independent rodent exposure in clustered ANDV-associated HCPS cases.

Household exposure was the most frequently reported P2P context in 15 studies [6,7,9,10,12,14,17,23,27,31,33,35,38,42,43], whereas healthcare-associated exposure was reported in a smaller subset of studies (*n* = 6) [7,10,17,27,28,43], either alone or in combination with household exposure.

#### 3.4.2. Incubation Period

Numerical incubation-period data were available only for a subset of studies. Reported values were generally concentrated around 2–4 weeks, with the widest ranges reported in studies of documented or strongly suspected P2P transmission. One zoonotic-exposure estimate of 42 days was retained descriptively but not interpreted as a P2P incubation estimate [34]. The most informative P2P estimates came from studies reporting either ranges for linked cases or individual-level transmission-pair data. All extracted incubation-period data are reported in Supplementary Table S3.

#### 3.4.3. Serial Interval

**Figure 3:**
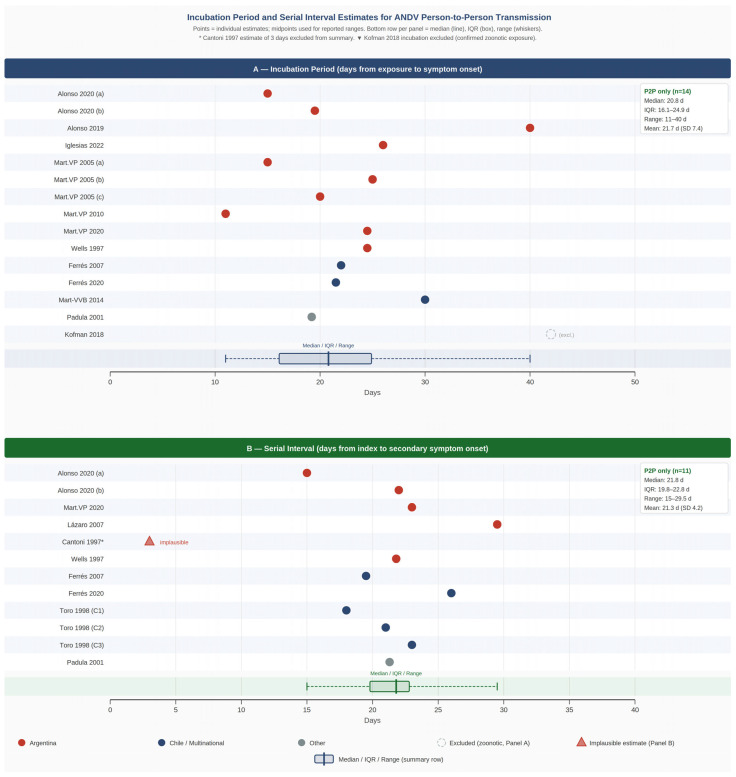
Descriptive summary of ANDV incubation period (Panel (**A**)) and serial interval (Panel (**B**)) estimates extracted across the included studies. Each point represents one extracted estimate; midpoint values were used for studies reporting ranges only. The vertical line denotes the median; the shaded box the IQR; and the whiskers the full observed range. Study labels indicate first author and year. Color indicates geographic origin: red = Argentina; blue = Chile; grey = Multinational. Dashed circle (Panel (**A**): Kofman 2018) [34] = estimate excluded from P2P pooled summary (confirmed zoonotic exposure). Triangle (Panel (**B**): Cantoni 1997*) [27] = biologically implausible estimate (3 days; see Section 3.4.3). Panel (**A**): Alonso 2020 [10]; Alonso 2019 [22]; Iglesias 2022 [33]; Martinez V.P. 2005 [9]; Martinez V.P. 2010 [38]; Wells 1997 [43]; Ferres 2007 [6]; Ferres 2020 [31]; Martinez Valdebenito 2014 [17]; Padula 2000 [3]; Kofman 2018 [34]. Panel (**B**): Alonso 2020 [10]; Martinez V.P. 2020 [7]; Lazaro 2007 [35]; Cantoni 1997 [27]; Wells 1997 [43]; Ferres 2007 [6]; Ferres 2020 [31]; Toro 1998 [42]; Padula 2000 [3].

Serial-interval data were reported in 10 studies [3,6,7,10,23,27,31,35,42,43]. Available estimates were mostly around 3 weeks, although individual values varied across clusters and study designs. Very short intervals, such as the 3-day value reported in one early outbreak investigation [27], should not be interpreted as biologically plausible P2P serial intervals and may reflect unrecognized common-source zoonotic exposure or uncertainty in symptom-onset reconstruction. Therefore, serial-interval estimates were summarized descriptively rather than pooled as formal meta-analytic estimates. The extracted incubation-period and serial-interval estimates are summarized in Figure 3, with detailed data reported in Supplementary Table S3.

#### 3.4.4. Secondary Attack Rate

Secondary attack rate (SAR) data amenable to quantitative extraction were identified in only three studies, precluding formal meta-analysis. Ferrés M. et al. [6], conducting a prospective cohort study, reported an overall household SAR of 3.4%, with marked stratification by contact type: 17.6% among sexual partners and 1.2% among non-sexual household contacts. This gradient is biologically consistent with documented viral RNA shedding in oral secretions [12], semen [15], and respiratory secretions and is consistent with a requirement for close, sustained mucosal exposure. Castillo C. et al. [28] documented a SAR of 0% among 20 contacts followed serologically, though the serosurvey design limited exclusion of prior immunity. Kofman A. et al. [34], involving a confirmed zoonotic index case, documented 0% SAR among 53 contacts, consistent with the absence of P2P spread in an exclusively zoonotic scenario.

### 3.5. Public Health and Outbreak Control Measures

Public health measures (Table 2) were inconsistently described; when reported, they mainly included contact tracing (*n* = 4) [6,14,33,35], isolation or infection-control measures (*n* = 3) [7,31,34], or multiple combined interventions. Hospital isolation in a specialized unit was documented in one maternal–neonatal transmission event [31]. PPE and infection control measures were reported in two studies involving healthcare worker transmission [27,28], consistently in the context of initial exposure prior to ANDV diagnosis.

The Chubut 2018–2019 outbreak [7] represented the largest and most extensively characterized P2P ANDV transmission event reported to date, with 34 confirmed cases and 33 documented secondary cases across multiple transmission generations. This outbreak provided evidence that, under specific epidemiological conditions, ANDV can sustain extended person-to-person transmission chains. Before control measures were implemented, the estimated reproductive number was reported to be above 2.0. Public health measures included temporary closure of a healthcare facility, contact tracing, quarantine of exposed individuals, reinforcement of personal protective equipment use, and revision of high-risk contact definitions.

### 3.6. Virological Findings

#### 3.6.1. Viral Detection, Viral Load, and Biological Compartments

**Figure 4:**
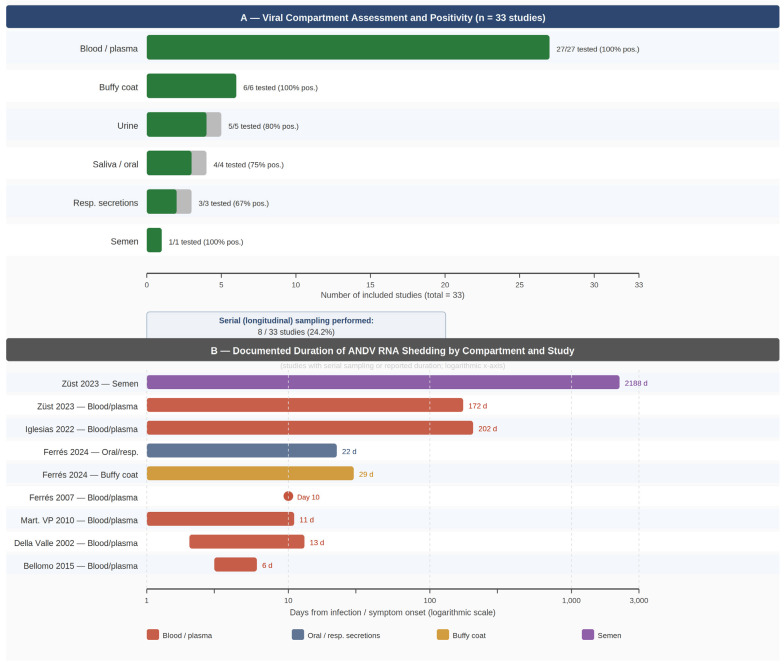
Viral shedding in ANDV infection. Panel (**A**): proportion of studies that assessed each biological compartment (grey background) and proportion of those studies that reported RNA positivity (green foreground). Proportions are calculated over studies that explicitly tested each compartment, not over all 33 included studies; numbers in parentheses indicate positive/tested counts. Panel (**B**): documented duration of ANDV RNA positivity by compartment and study, plotted on a logarithmic time axis (days from infection or symptom onset). Bars represent the reported or estimated detection window; circles indicate a single documented timepoint. Color indicates compartment: red = blood/plasma; blue = oral/respiratory; amber = buffy coat; purple = semen. Zust 2023 [15]; Iglesias 2022 [33]; Ferres 2024 [12]; Ferres 2007 [6]; Martinez V.P. 2010 [38]; Della Valle 2002 [30]; Bellomo 2015 [25].

Virological data were reported across all 33 included studies (Table 3 and Figure 4, panel A). ANDV RNA detection by RT-PCR or RT-qPCR was reported as positive in 28 studies, whereas 5 studies [26,28,29,41,43] did not report RNA detection.

Blood or plasma was the most consistently sampled compartment, confirmed positive in all studies expect six studies [13,26,28,29,41,43], reporting a blood compartment result (100%). Quantitative viral load data were reported in seven studies [6,7,15,23,25,33,37]. The highest documented mean blood viral load was 5 × 10^7^ copies/mL, reported during the Chubut 2018–2019 outbreak [7]. Buffy coat viral loads were reported by Lopez et al. (range 8.2 × 10^3^ to 2.4 × 10^5^ copies/mL) [37]. Semen viral load was quantified by Zust R. et al. [15] at approximately 2.7 × 10^4^ copies/mL.

Isolation of replication-competent virus by cell culture was confirmed in five studies [3,12,13,22,30], with an additional four studies detecting RNA without culture confirmation [7,17,23,42]. In all other studies, culture-based isolation was not attempted or not reported. This critical gap limits the capacity to draw conclusions about in vivo infectiousness or transmissibility from RNA data alone.

Extravascular compartments were assessed in a minority of studies. Saliva and oral fluids were tested in four studies [12,13,31,32]. Urine was positive in three studies [12,15,31]. Respiratory secretions were positive in two studies [3,23]. Breast milk positivity was documented in one maternal–neonatal transmission event [31]. As the large majority of studies did not systematically assess extravascular compartments, these proportions reflect testing practices rather than true population-level shedding frequencies.

#### 3.6.2. Duration and Kinetics of Viral Shedding

Serial sampling was performed in only 10 of 33 studies [3,6,12,14,15,22,25,31,32,33] (Figure 4B). The longest documented ANDV RNA shedding duration was reported by Zust R. et al. [15]: viral RNA remained detectable in semen for 2188 days (71 months) post-infection, with blood viraemia clearing at 172 days post-symptom onset and replication-competent virus successfully isolated from semen samples collected years after acute infection. Iglesias A.A. et al. [33] documented detectable RNA in peripheral blood up to 202 days post-fever onset. Ferrés M. et al. [12] documented oral and respiratory fluid positivity up to 22 days post-symptom onset, with buffy coat RNA persistence beyond the 29-day follow-up and successful culture-based infectivity assays in RT-qPCR-positive gingival crevicular fluid, saliva, nasopharyngeal swab, and urine samples obtained during the acute phase.

Prolonged detection of ANDV RNA, and in selected reports replication-competent virus, in immune-privileged compartments such as semen may have implications for post-discharge counselling and case management. However, the available evidence remains insufficient to define the magnitude or duration of sexual transmission risk in convalescent individuals.

### 3.7. Risk-of-Bias Assessment

**Figure 5:**
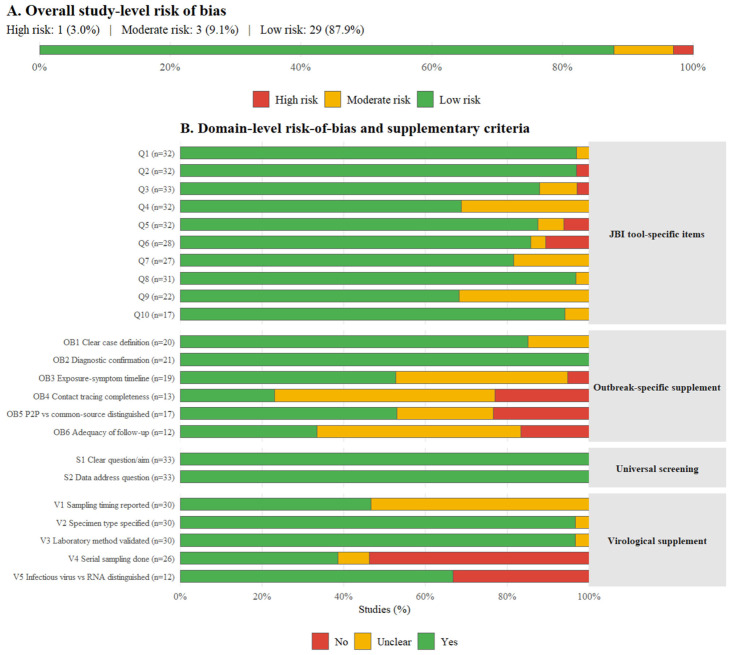
Risk-of-bias assessment of the included studies. Panel (**A**) shows the overall study-level risk-of-bias classification. Panel (**B**) summarizes item-level responses across JBI tool-specific items, outbreak-specific supplementary domains, universal screening items, and virological supplementary domains.

Risk of bias (Figure 5) was assessed for all 33 studies using the JBI tool matched to study design, supplemented by outbreak-specific (OB1–OB6) and virological (V1–V5) domains. All studies were classified as low risk of bias, except for three (9.1%) classified as moderate risk [17,26,32] and one (3.0%) as high risk [30]. The mean JBI score was 86.9% (median 90.0%; range 40–100%). The overall risk-of-bias judgement reflected the general methodological quality of each study according to the applicable JBI checklist and supplementary domains; it should not be interpreted as equivalent to high certainty for all transmission-specific outcomes. Uncertainty in transmission attribution was assessed separately, particularly regarding contact tracing completeness, reconstruction of exposure-to-symptom timelines, and exclusion of shared zoonotic exposure. Consequently, studies with otherwise-adequate reporting could still provide only supportive evidence when person-to-person transmission remained uncertain.

Overall, the risk-of-bias assessment indicated generally adequate reporting of diagnostic and laboratory domains, while the main limitations concerned outbreak reconstruction and virological timing. Diagnostic confirmation, specimen description, and laboratory methods were usually clearly reported, with only isolated exceptions. By contrast, several outbreak-specific domains were less consistently addressed, particularly completeness of contact tracing, reconstruction of exposure-to-symptom timelines, and the ability to distinguish P2P transmission from shared zoonotic exposure. For virological studies, the most frequent limitation was incomplete reporting of sampling time relative to symptom onset, together with limited use of serial sampling.

These domain-level gaps have direct implications for the reliability of transmission parameters reported in Section 3.4 and Section 3.5. Low completeness of OB4 implies that SAR denominators are likely underestimated due to incomplete contact ascertainment. Low OB5 completeness means that P2P classifications in many studies may be confounded by unrecognized independent zoonotic exposure. The high proportion of unclear V1 ratings means that the temporal relationship between viral positivity and clinical stage cannot be reliably characterized across most virological studies.

Accordingly, findings supported by laboratory confirmation, clear epidemiological linkage, and/or molecular evidence were considered more robust. Findings from studies with incomplete contact tracing, uncertain exposure reconstruction, possible shared zoonotic exposure, or limited sampling information were retained but interpreted as supportive rather than definitive.

When interpreted according to transmission certainty, the evidence for human-to-human transmission of ANDV remained supported by studies with documented epidemiological and/or molecular linkage. However, estimates of secondary attack rate, incubation period, and serial interval were interpreted more cautiously when derived from suspected or mixed-exposure settings.

## 4. Discussion

### 4.1. Principal Findings

This systematic review identified 33 eligible studies published between 1997 and 2025, encompassing 17,204 individuals and 2221 laboratory-confirmed ANDV cases. P2P transmission was classified as the primary or co-occurring route in 20 studies (60.6%), with virological or robust epidemiological documentation in 6. The descriptive pooled incubation period was median 20.8 days (IQR 16.1–24.9; range 11–40 days), and the serial interval median was 21.8 days (IQR 19.8–22.8 days); formal meta-analysis was precluded by clinical and methodological heterogeneity across study designs and outcome definitions. Secondary attack rate was numerically extractable in only three studies, ranging from 0% among non-sexual household contacts to 17.6% among sexual partners. ANDV RNA was detected in 100% of blood specimens tested across all reporting studies; isolation of replication-competent virus was confirmed in four studies, with additional positivity documented in saliva, urine, respiratory secretions, and semen. The longest documented shedding duration was 2188 days in semen. Risk of bias was low in 87.9% of studies by overall JBI assessment, though the outbreak-specific domains of contact tracing completeness and P2P versus zoonotic source attribution were systematically deficient.

### 4.2. Interpretation and Comparison with the Existing Literature

To our knowledge, this is the first systematic review specifically and exclusively addressing P2P transmission dynamics, viral shedding, and public health management of ANDV, and the first to adopt a pre-registered protocol with domain-level outbreak-specific and virological risk-of-bias assessment. The 2022 systematic review by Toledo et al. [44] examined human-to-human hantavirus transmission across all species (22 studies, search cutoff February 2021) and concluded that the balance of evidence from comparative designs did not firmly support it, largely due to the inability to exclude co-rodent exposure as a confounder. The present review extends and updates previous work: it is ANDV-specific, incorporates 11 additional studies, including several published after 2021 that add important new evidence, performs disease-specific risk-of-bias assessment, and, crucially, provides the first quantitative synthesis of incubation period, serial interval, and secondary attack rate for ANDV. Together, full-length genomic evidence of P2P spread, prospective culture-confirmed multi-compartment shedding, and cohort-level transmission data strengthen the evidence base compared with that available at the time of the previous review. The pooled incubation period (median 20.8 days, range 11–40) confirms and refines earlier individual-study estimates. The 40-day upper bound suggests that 30-day follow-up periods may fail to capture a minority of late-onset cases, supporting the precautionary 42-day monitoring or quarantine period adopted in recent international guidance for high-risk contacts [7]. The serial interval (median 21.8 days, IQR 19.8–22.8) closely approximates the incubation period and is notably narrow, consistent with infectiousness concentrated around symptom onset, a pattern supported by the pre-symptomatic viraemia documented by Ferrés et al. [6], who detected ANDV RNA in blood 5–15 days before any clinical presentation.

The near-15-fold SAR differential between sexual partners (17.6%) and non-sexual household contacts (1.2%) [6] is the most informative quantitative transmission indicator available and is mechanistically coherent with the virological evidence from Ferrés et al. [12], who isolated replication-competent ANDV from gingival crevicular fluid, saliva, and respiratory secretions, in 131 confirmed cases, and with the ultrastructural localization of ANDV antigen to salivary gland excretory pathways by Pizarro et al. [13]. These findings support the hypothesis that ANDV P2P transmission is more likely to occur after close mucosal or respiratory exposure than through efficient long-range airborne spread, although direct comparative evidence on transmission routes remains limited.

The documentation by Züst et al. [15] of replication-competent ANDV in semen months post-infection, with RNA persistence for as long as 71 months, is unprecedented in the hantavirus literature. It draws a direct parallel with Ebola virus disease, where post-convalescent seminal shedding led to confirmed secondary sexual transmissions and ultimately prompted WHO-level revision of discharge protocols. No confirmed post-convalescent ANDV sexual transmission has been published, but no included study was designed to detect it, so this is a gap of investigative design, not of biological implausibility.

The Chubut 2018–2019 outbreak [7] established that ANDV can sustain amplified multi-generation P2P transmission chains with a reproductive number exceeding 2.0, driven by individuals with blood viral loads of 5 × 10^7^ copies/mL. This directly challenges the historical assumption that ANDV P2P spread is inherently self-limiting to small household clusters and suggests that viral load may contribute to individual-level infectiousness, although evidence remains insufficient to define viral-load thresholds for isolation prioritization.

### 4.3. Implications for Public Health and Clinical Practice

The findings of this systematic review suggest several provisional public health considerations for outbreak preparedness, clinical management, and infection prevention policy, based on the best available but still limited evidence. Given the predominantly observational and heterogeneous nature of the evidence base, these considerations should be interpreted as precautionary implications that may require adaptation to local epidemiology, exposure context, health-system capacity, and evolving evidence. The strength and directness of evidence also varied across these considerations. Prolonged monitoring and risk-stratified contact tracing are supported mainly by direct ANDV-specific epidemiological evidence from outbreak investigations and contact studies, whereas post-discharge counselling regarding possible sexual transmission relies primarily on limited virological evidence, biological plausibility, and analogy with other high-consequence viral infections. Accordingly, these implications should not be interpreted as having the same level of evidentiary certainty.

Their relevance extends well beyond the endemic setting, as demonstrated by travel-associated cases managed in Switzerland [15] and the 2026 multinational MV Hondius cruise ship outbreak, which prompted WHO emergency guidance recommending active monitoring and quarantine of high-risk contacts for 42 days [45]. These findings indicate that ANDV should be considered in international outbreak preparedness and high-consequence infectious disease planning, particularly given the occurrence of travel-associated cases [46,47]. Health-system capacity and institutional factors are likely to influence the ability to translate these recommendations into effective outbreak response [48].

The near-15-fold differential in secondary attack rate between sexual partners (17.6%) and non-sexual household contacts (1.2%) [6] supports consideration of risk-stratified contact follow-up, particularly where exposure type and intensity can be clearly documented.

This approach is well established for pathogens with documented contact-type-specific transmission gradients, including SARS-CoV-2 and Ebola virus disease, where stratification has improved response efficiency without overburdening low-risk contacts [49,50]. No validated ANDV-specific contact risk stratification algorithm currently exists; its development and prospective validation would help translate these observations into operational guidance [51].

Healthcare worker transmission was documented or suspected in at least six included studies across three decades, consistently in the context of unprotected exposure before ANDV diagnosis [6,7,17]. Culture-confirmed infectious virus in gingival crevicular fluid, saliva, and respiratory secretions during the acute phase [12] supports a precautionary rationale for considering mucosal and respiratory barrier precautions, including respirators, eye protection, and gloves, from the point of clinical suspicion, before laboratory confirmation, consistent with ECDC guidance issued in May 2026 (ECDC Rapid Scientific Advice). However, no included study prospectively evaluated the effectiveness of specific infection prevention measures for ANDV, and the optimal PPE approach remains uncertain.

The detection of replication-competent ANDV in semen months post-infection, with RNA persistence for up to 71 months [15], raises a provisional concern for post-discharge counselling, but the evidence remains indirect. Unlike Ebola virus disease [52], for which post-convalescent sexual transmission has been documented and incorporated into WHO guidance, no confirmed ANDV sexual transmission chain has been established in convalescent individuals. Therefore, any post-discharge sexual counselling for ANDV should be framed as precautionary and based on limited virological evidence, biological plausibility, and analogy with other high-consequence viral infections, rather than as definitive ANDV-specific guidance.

As highlighted by Vial et al. [53], clinical management guidance for ANDV remains inadequate relative to its severity and case-fatality rate.

ANDV combines a case-fatality rate approaching 40%, confirmed P2P transmissibility, and the absence of any licensed vaccine or proven antiviral therapy. The evidence synthesized in this review, including incubation period, serial interval, exposure types, and virological findings, may inform clinical recognition criteria, diagnostic algorithms, and case-management protocols for healthcare providers in non-endemic settings [54], while acknowledging that some parameters remain based on limited and heterogeneous evidence.

Consideration of ANDV within standing high-consequence infectious disease (HCID) preparedness frameworks in Europe, North America, and other regions with documented imported cases may be warranted [55].

More broadly, the epidemiological parameters synthesized in this review represent the foundational inputs for next-generation outbreak forecasting and contact management tools [56]. Emerging applications of machine learning and artificial intelligence in infectious disease diagnosis, risk prediction, and post-discharge surveillance offer a promising framework for translating this evidence base into operational decision-support systems, including early-warning algorithms and automated contact risk stratification tools, as has been demonstrated for other high-consequence infectious diseases [57,58,59,60,61].

### 4.4. Strengths and Limitations

This review has several methodological strengths. The protocol was prospectively registered, and the review was conducted and reported in accordance with PRISMA, with data synthesis following the Swim guideline. It is the first systematic review exclusively focused on ANDV P2P transmission, covering the complete 1997–2025 evidence base across 33 studies, with risk-of-bias assessment extended by two purpose-built supplementary domain sets and independent four-reviewer data extraction. Several limitations must be acknowledged. The geographical concentration of the evidence base, with 88% of studies from Chile and Argentina, limits generalizability to other ANDV lineages and settings. This limitation may have been compounded by the restriction to English-language publications, which could have excluded relevant studies, surveillance reports, outbreak investigations, or public health documents published in Spanish or Portuguese from endemic Latin American regions. In addition, the predominance of studies from high-income and upper-middle-income countries should be interpreted as a feature of the available evidence base rather than as evidence that ANDV transmission risk is concentrated in higher-income settings. This pattern likely reflects the concentration of published ANDV research in Chile and Argentina, as well as differences in surveillance systems, laboratory diagnostic capacity, outbreak investigation infrastructure, genomic confirmation, and publication practices. Because income level was assigned at the country level, it may not capture subnational rural vulnerability, healthcare access, or local ecological risk. Formal meta-analysis was not feasible; all epidemiological parameter estimates are descriptive syntheses, and midpoint imputation for reported ranges introduces imprecision that cannot be quantified. Therefore, inferences regarding incubation period, serial interval, and secondary attack rate should be made cautiously. The secondary attack rate was numerically extractable in only three studies and is almost certainly underestimated given the systematic deficiency in contact tracing completeness documented across the corpus. Most included studies used non-comparative observational designs with an inherent inability to fully exclude concurrent zoonotic co-exposure. No included study was designed to detect asymptomatic infection, pre-symptomatic transmission, or convalescent sexual transmission. In addition, detection of ANDV RNA by RT-PCR should not be interpreted as equivalent to infectious viral shedding. Molecular positivity demonstrates the presence of viral genetic material in a given specimen but does not establish replication-competent virus, infectiousness, or transmissibility. Because culture-confirmed isolation of infectious ANDV from human specimens has been reported only rarely, RNA detection across biological matrices was interpreted cautiously as evidence of compartmental viral presence and biological plausibility, rather than as direct evidence of infectious shedding or transmission risk. Finally, publication bias towards positive transmission events cannot be excluded. Although this review cannot establish why ANDV, unlike other hantaviruses, is capable of person-to-person transmission, the findings support a biologically plausible link between close-contact transmission and viral presence in compartments relevant to mucosal or respiratory exposure. This interpretation remains provisional, because RNA detection alone does not prove infectious shedding and comparative studies with other hantaviruses are lacking. This review is also limited by the heterogeneity of the included evidence. Because documented person-to-person transmission of ANDV is rare, the available literature includes diverse study designs with variable methodological rigor and completeness of exposure reconstruction. Therefore, lower-level or indirect evidence was interpreted cautiously and used mainly as contextual or supportive information. The main conclusions rely primarily on studies with epidemiologically documented or genomically supported person-to-person transmission.

## 5. Conclusions

This systematic review confirms that Andes virus differs from other hantaviruses in its documented capacity for person-to-person transmission, particularly after close physical, household, healthcare, or intimate contact. However, the strength of evidence varies across studies, and source attribution is often limited by incomplete contact tracing and difficulty excluding shared zoonotic exposure. The available data suggest an incubation period and serial interval of approximately three weeks, with reported upper bounds close to 40 days, although these values should be interpreted as exploratory descriptive summaries rather than pooled estimates. These findings support consideration of prolonged follow-up of high-risk contacts and careful alignment of monitoring or quarantine periods with the observed incubation range. Virological evidence shows that ANDV RNA can be detected in blood and, less consistently, in saliva, respiratory secretions, urine, and semen. Culture-confirmed virus isolation from selected compartments supports the biological plausibility of transmission through close mucosal or respiratory exposure, although RNA detection alone should not be interpreted as proof of infectiousness. Overall, the public health implications of this review should be interpreted according to the strength and directness of the underlying evidence: prolonged monitoring and risk-stratified contact tracing are supported mainly by ANDV-specific epidemiological evidence, whereas more indirect considerations, such as post-discharge counselling regarding possible sexual transmission, remain provisional and require further evidence. ANDV should be considered in outbreak preparedness, infection prevention, contact tracing, and high-consequence infectious disease planning, including non-endemic regions with potential for imported cases. Future research should prioritize prospective contact studies, standardized exposure definitions, genomic confirmation of transmission chains, and serial multi-compartment sampling.

## Figures and Tables

**Table 1:** Characteristics of the included studies.

First Author (Year)	Country	Study Period	Study Design	Income Level	Geographic Scope	N Total Cases	N Confirmed ANDV	N Secondary Cases	Reservoir Data
Alonso D.O. (2019) [22]	Argentina	2009– 2017	Virological study	U-M	National surveillance	4.488	533	NR	Yes—Oligoryzomys longicaudatus
Alonso D.O. (2020) [10]	Argentina	2014	Outbreak investigation	U-M	Single outbreak/cluster	5	5	2	Yes—other rodent
Alonso D.O. (2024) [23]	Argentina	1995– 2022	Virological study	U-M	Regional surveillance	934	NR	NR	NR
Barrera A. (2025) [14]	Chile	May– June 2024	Virological study	H	Single outbreak/cluster	4	2	2	NR
Bellomo C.M. (2015) [25]	Argentina	2011	Virological study	U-M	Regional surveillance	958	73	0	NR
Bellomo C.M. (2025) [24]	Argentina	July 2024	Virological study	U-M	Single case (sporadic)	1	1	0	Yes—Oligoryzomys longicaudatus
Busch M. (2004) [26]	Argentina	January 1998–December 2001	Ecological/geographic	U-M	Regional surveillance	85	85	NR	NR
Cantoni G. (1997) [27]	Argentina	1993– 1996	Outbreak investigation	U-M	Single outbreak/cluster	26	NR	NR	Yes—Oligoryzomys longicaudatus
Castillo C. (2000) [28]	Chile	1997– 1999	Seroprevalence/serosurvey	H	Regional surveillance	20	20	0	Yes—Oligoryzomys longicaudatus
Castillo C. (2001) [29]	Chile	1997 and 1999	Case series	H	Single outbreak/cluster	16	16	NR	Yes—other rodent
Della Valle M.G. (2002) [30]	Argentina	1997– 1999	Virological study	U-M	Regional surveillance	20	20	NR	No rodent data
Ferrés M. (2007) [6]	Chile	November 2001–June 2005	Cohort prospective	H	Multiple outbreaks	92	92	16	Yes—Oligoryzomys longicaudatus
Ferrés M. (2020) [31]	Chile	/	Case report	H	Single case (sporadic)	2	2	1	No rodent data
Ferrés M. (2024) [12]	Chile	2008–2022	Cohort prospective	H	National surveillance	131	131	21	Yes—other rodent
Godoy P. (2009) [32]	Chile	2000– 2002	Cohort prospective	H	Multiple outbreaks	52	52	NR	Yes—Oligoryzomys longicaudatus
Iglesias A.A. (2022) [33]	Argentina	2014– 2019	Cohort prospective	U-M	Multiple outbreaks	441	154	38	Yes—Oligoryzomys longicaudatus
Kofman A. (2018) [34]	USA	January–March 2018	Outbreak investigation	H	Single case (sporadic)	1	1	0	Yes—Oligoryzomys longicaudatus
Lázaro M.E. (2007) [35]	Argentina	1993–2005	Outbreak investigation	U-M	Multiple outbreaks	51	15	10	NR
López R. (2019) [36]	Chile	2001– 2018	Cohort retrospective	H	National surveillance	175	175	NR	Yes—Oligoryzomys longicaudatus
Lopez R. (2021) [37]	Chile	February–December 2017	Case series	H	Single outbreak/cluster	5	5	NR	Yes—unspecified rodent
Martinez V.P. (2005) [9]	Argentina	July–December 2002	Outbreak investigation	U-M	Multiple outbreaks	13	13	7	No rodent data
Martinez V.P. (2010) [38]	Argentina	1995– 2008	Case series	Mixed	National surveillance	8.522	710	NR	Yes—Oligoryzomys longicaudatus
Martinez V.P. (2020) [7]	Argentina	November 2018–February2019	Outbreak investigation	U-M	Single outbreak/cluster	34	34	33	Yes—unspecified rodent
Martinez-Valdebenito C. (2014) [17]	Chile	February–April 2011	Outbreak investigation	U-M	Single outbreak/cluster	5	5	0	No rodent data
Muñoz-Zanzi C. (2015) [39]	Chile	1995– 2014	Seroprevalence/serosurvey	U-M	Regional surveillance	934	12	NR	No rodent data
Padula P. (2002) [40]	Bolivia	2000	Case report	L-M	Single case (sporadic)	1	1	NR	Yes—Oligoryzomys longicaudatus
Padula P.J. (2001) [3]	Multinational	1995– 1999	Case series	Mixed	Multinational	87	NR	NR	NR
Pizarro E. (2020) [13]	Chile	1999– 2004	Virological study	H	Single outbreak/cluster	10	10	NR	NR
Riquelme R. (2003) [41]	Chile	1997– 2001	Case series	U-M	Regional surveillance	25	25	NR	NR
Toro J. (1998) [42]	Chile	July 1997–January 1998	Outbreak investigation	H	Single outbreak/cluster	25	16	5	NR
Vial P.A. (2006) [11]	Chile	NR	Case series	H	Single outbreak/cluster	20	12	NR	Yes—Oligoryzomys longicaudatus; Akodon longipilis; Abrothix
Wells R. (1997) [43]	Argentina	September–December 1996	Outbreak investigation	U-M	Single outbreak/cluster	20	NR	NR	Yes—other rodent (Abrothrix longipilis)
Zust R. (2023) [15]	Switzerland	December 2016	Virological study	H	Single case (sporadic)	1	1	NR	NR

ANDV = Andes orthohantavirus. Income: H = High; U-M = Upper-Middle; L-M = Low-Middle. Geographic scope: single cluster = a defined outbreak or case cluster; national/regional surveillance = population-based or multi-year studies. N Secondary Cases: documented secondary ANDV cases identified through contact tracing or follow-up; NR = not reported.

**Table 2:** Summary of included studies reporting clinical diagnosis, laboratory confirmation, specimen type, viral RNA detection and key transmission/public health variables.

First Author (Year)	Clinical Syndrome Reported	ANDV Confirmation	Specimen Type	Blood Positive	Viral RNA Positive (Copies/mL)	Transmission Type	P2P Context	PH Measures Adopted
Alonso D.O. (2019) [22]	Yes—ANDV confirmed	PCR + serology	BP	Yes	NR	P2Ps	NR	NR
Alonso D.O. (2020) [10]	Yes—ANDV confirmed	PCR + serology	BP	Yes	Yes	P2Pd	HH + HCW	NR
Alonso D.O. (2024) [23]	Yes—ANDV confirmed	PCR confirmed	BP	Yes	Yes	P2Ps + Z	HH	NR
Barrera A. (2025) [14]	Yes—ANDV confirmed	PCR + serology	MS (serum, plasma, buffy coat)	Yes	Yes	P2Ps	HH	CT
Bellomo C.M. (2015) [25]	Yes—ANDV confirmed	PCR + serology	BP	Yes	Yes (median 2.7 + 10^6^ RNA copy numbers)	NR	NR	NR
Bellomo C.M. (2025) [24]	Yes—ANDV confirmed	PCR + serology	Serum	Yes	Yes	Z	NR	NR
Busch M. (2004) [26]	Yes—HPS/HCPS unspecified	NR	NR	NR	NR	Z	NR	NR
Cantoni G. (1997) [27]	Yes—HPS/HCPS unspecified	Serology confirmed (IgM/IgG)	BP	Yes	Yes	P2Ps	HH + HCW	PPE + IC
Castillo C. (2000) [28]	Yes—ANDV confirmed	Serology confirmed (IgM/IgG)	Serum	NR	NR	P2Ps	HCW	PPE + IC
Castillo C. (2001) [29]	Yes—ANDV confirmed	Serology confirmed (IgM/IgG)	Serum	NR	NR	Z	NR	NR
Della Valle M.G. (2002) [30]	Yes—ANDV confirmed	PCR + serology	BP	Yes	Yes	Z	NR	NR
Ferrés M. (2007) [6]	Yes—ANDV confirmed	PCR + serology	BP	Yes	Yes (Case 16: 2.8 × 10^3^ Case 15: 3.3 × 10^5^ Case 13: 1.8 × 10^5^ Case 10: 1.1 × 10^6^ Case 14: 1.2 × 10^5^ Case 9: 3.9 × 10^3^)	P2P + Z	HH	CT
Ferrés M. (2020) [31]	Yes—ANDV confirmed	PCR + serology	MS (blood, saliva, urine, breast milk)	Yes	Yes	P2Pd	HH	HI
Ferrés M. (2024) [12]	Yes—ANDV confirmed	PCR + serology	MS (buffy coat, blood, saliva, respiratory swab, urine, gingival cervical fluid)	Yes	Yes	P2Ps	HH	NR
Godoy P. (2009) [32]	Yes—ANDV confirmed	PCR + serology	Urine	Yes	Yes	P2Ps	NR	NR
Iglesias A.A. (2022) [33]	Yes—ANDV confirmed	PCR + serology	MS	Yes	Yes (4.1 × 10^3^)	P2Pd + Z	HH	CT
Kofman A. (2018) [34]	Yes—ANDV confirmed	PCR + serology	BP	Yes	Yes	Z	NR	MM
Lázaro M.E. (2007) [35]	Yes—ANDV confirmed	PCR + serology	BP	Yes	Yes	P2Pd	HH	CT
López R. (2019) [36]	Yes—ANDV confirmed	PCR + serology	BP	Yes	Yes	NR	NR	NR
Lopez R. (2021) [37]	Yes—ANDV confirmed	PCR + serology	Buffy coat	Yes	Yes (Patient 1: 2.4 × 10^5^; Patient 2: 2.7 × 10^4^; Patient 3: 8.2 × 10^3^; Patient 4: 4.1 × 10^4^)	NR	NR	NR
Martinez V.P. (2005) [9]	Yes—ANDV confirmed	PCR + serology	BP	Yes	Yes	P2P + Z	HH	NR
Martinez V.P. (2010) [38]	Yes—ANDV confirmed	PCR + serology	BP	Yes	NR	P2Ps	HH	NR
Martinez V.P. (2020) [7]	Yes—ANDV confirmed	PCR + serology	BP	Yes	Yes (1.7 × 10^5^–1.3 × 10^8^, mean 5 × 10^7^, median 4.4 × 10^7^)	P2Pd	HH + HCW	MM
Martinez-Valdebenito C. (2014) [17]	Yes—ANDV confirmed	PCR + serology	BP	Yes	Yes	P2Ps	HH + HCW	NR
Muñoz-Zanzi C. (2015) [39]	Yes—ANDV confirmed	PCR + serology	BP	Yes	Yes	NR	NR	NR
Padula P. (2002) [40]	Yes—ANDV confirmed	PCR + serology	BP	Yes	Yes	NR	NR	NR
Padula P.J. (2001) [3]	Yes—HPS/HCPS unspecified	PCR + serology	MS (blood/serum/clot organ)	Yes	Yes	P2Pd	NR	NR
Pizarro E. (2020) [13]	Yes—ANDV confirmed	Serology confirmed (IgM/IgG)	MS (saliva, submandibular salivary gland tissue, lung tissue post mortem, salivary gland excretory pathway/lumen)	NR	NR	P2Ps	NR	NR
Riquelme R. (2003) [41]	Yes—ANDV confirmed	Serology confirmed (IgM/IgG)	Serum	NR	NR	Z	NR	NR
Toro J. (1998) [42]	Yes—ANDV confirmed	PCR + serology	BP	Yes	Yes	P2Ps	HH	NR
Vial P.A. (2006) [11]	Yes—ANDV confirmed	PCR + serology	BP	Yes	Yes	Z	NR	NR
Wells R. (1997) [43]	Yes—ANDV confirmed	PCR + serology	MS (NR)	NR	Yes	P2Ps	HH + HCW	NR
Zust R. (2023) [15]	Yes—ANDV confirmed	PCR + serology	MS (blood, semen)	Yes	Yes (semen: 26.7 × 10^3^; supernatant 30 × 10^3^; resuspended cell pellet 27 × 10^3^)	NR	NR	NR

ANDV = Andes virus; BP = blood/plasma; CT = contact tracing; HCPS = hantavirus cardiopulmonary syndrome; HCW = healthcare worker exposure; HH = household/family contact; HI = hospital isolation; HPS = hantavirus pulmonary syndrome; IC = infection control; MM = multiple measures; MS = multiple specimen type; NR = not reported; P2P = person-to-person transmission; P2Pd = person-to-person documented; P2Ps = person-to-person suspected; PH = public health; PPE = personal protective equipment; Z = zoonotic transmission/rodent exposure. Transmission categories were harmonized according to certainty of attribution: genomic confirmation, epidemiologically documented P2P transmission, suspected/probable P2P transmission, and mixed/uncertain exposure.

**Table 3:** Virological characteristics by study: specimen type, viral RNA detection, quantitative viral load, compartmental positivity, serial sampling, and documented shedding duration.

First Author (Year)	Specimen Type	RNA Positive	Viral Load (Copies/mL)	Blood+	Saliva+	Urine+	Respiratory+	Serial Sampling	Shedding Duration	Infectious Virus Isolated
Alonso D.O. (2019) [22]	Blood/plasma	Yes	NR	Yes	NR	NR	NR	Yes	NR	Yes—culture confirmed
Alonso D.O. (2020) [10]	Blood/plasma	Yes	Yes	Yes	NR	NR	NR	NR	NR	Not assessed
Alonso D.O. (2024) [23]	Blood/plasma	Yes	Mean 5 × 10^7^ copies/mL	Yes	NR	NR	Yes	NR	NR	No—RNA only
Barrera A. (2025) [14]	Multiple (serum, plasma, buffy coat)	Yes	NR	Yes	NR	NR	NR	Yes	NR	Not assessed
Bellomo C.M. (2015) [25]	Blood/plasma	Yes	Median 2.7 × 10^6^ copies/mL	Yes	NR	NR	NR	Yes	Days 3–6	Not assessed
Bellomo C.M. (2025) [24]	Serum	Yes	Yes	Yes	NR	NR	NR	No	NR	Not assessed
Busch M. (2004) [26]	NR	NR	NR	NR	NR	NR	NR	No	NR	Not assessed
Cantoni G. (1997) [27]	Blood/plasma	Yes	Yes	Yes	NR	NR	NR	No	NR	Not assessed
Castillo C. (2000) [28]	Serum	NR	NR	NR	NR	NR	NR	No	NR	Not assessed
Castillo C. (2001) [29]	Serum	NR	NR	NR	NR	NR	NR	No	NR	Not assessed
Della Valle M.G. (2002) [30]	Blood/plasma	Yes	Yes	Yes	NR	NR	NR	No	Days 2–13	Yes—culture confirmed
Ferrés M. (2007) [6]	Blood/plasma	Yes	3.9 × 10^3^–1.2 × 10^6^ copies/mL	Yes	NR	NR	NR	Yes	NR	Not assessed
Ferrés M. (2020) [31]	Multiple (blood, saliva, urine, breast milk)	Yes	Yes	Yes	Yes	Yes	NR	Yes	NR	Not assessed
Ferrés M. (2024) [12]	Multiple (buffy coat, blood, saliva, resp. swab, urine, GCF)	Yes	Yes	Yes	Yes	Yes	Yes	Yes	Oral ≤ 22 d; buffy coat ≥ 29 d	Yes—culture confirmed
Godoy P. (2009) [32]	Urine	Yes	Yes	Yes	Yes	NR	NR	Yes	NR	Not assessed
Iglesias A.A. (2022) [33]	Multiple	Yes	4.1 × 10^3^ copies/mL	Yes	NR	NR	NR	Yes	≤202 d (blood)	Not assessed
Kofman A. (2018) [34]	Blood/plasma	Yes	Yes	Yes	NR	NR	NR	No	NR	Not assessed
Lázaro M.E. (2007) [35]	Blood/plasma	Yes	Yes	Yes	NR	NR	NR	No	NR	Not assessed
López R. (2019) [36]	Blood/plasma	Yes	Yes	Yes	NR	NR	NR	No	NR	Not assessed
Lopez R. (2021) [37]	Buffy coat	Yes	2.4 × 10^5^–8.2 × 10^3^ copies/mL	Yes	NR	NR	NR	No	NR	Not assessed
Martinez V.P. (2005) [9]	Blood/plasma	Yes	Yes	Yes	NR	NR	NR	No	NR	Not assessed
Martinez V.P. (2010) [38]	Blood/plasma	Yes	NR	Yes	NR	NR	NR	No	Days 1–11	NR
Martinez V.P. (2020) [7]	Blood/plasma	Yes	1.7 × 10^5^–1.3 × 10^8^ (mean 5 × 10^7^)	Yes	NR	NR	NR	No	NR	No—RNA only
Martinez-Valdebenito C. (2014) [17]	Blood/plasma	Yes	Yes	Yes	NR	NR	No	No	NR	No—RNA only
Muñoz-Zanzi C. (2015) [39]	Blood/plasma	Yes	Yes	Yes	NR	NR	NR	No	NR	Not assessed
Padula P. (2002) [40]	Blood/plasma	Yes	Yes	Yes	NR	NR	NR	No	NR	Not assessed
Padula P.J. (2001) [3]	Multiple (blood, serum, clot, organ)	Yes	Yes	Yes	NR	NR	Yes	Yes	NR	Yes—culture confirmed
Pizarro E. (2020) [13]	Multiple (saliva, salivary gland, lung, GCF)	Yes	NR	NR	Yes	NR	NR	No	NR	Yes—culture confirmed
Riquelme R. (2003) [41]	Serum	NR	NR	NR	NR	NR	NR	No	NR	Not assessed
Toro J. (1998) [42]	Blood/plasma	Yes	Yes	Yes	NR	NR	NR	No	NR	No—RNA only
Vial P.A. (2006) [11]	Blood/plasma	Yes	Yes	Yes	NR	NR	NR	No	NR	Not assessed
Wells R. (1997) [43]	Multiple (NR)	NR	Yes	NR	NR	NR	NR	No	NR	Not assessed
Zust R. (2023) [15]	Multiple (blood, semen)	Yes	Semen: 2.7 × 10^4^ copies/mL	Yes	NR	Yes	NR	Yes	Semen: 2188 d; blood: ≤ 172 d	Not assessed

Note. RNA Positive: Yes = ANDV RNA detected by RT-PCR or RT-qPCR; NR = not reported. Viral Load: quantitative copies/mL where available; Yes = RNA detected but not quantified; NR = not reported or not performed. Blood/Saliva/Urine+: positivity in that compartment among studies that tested it; NR = compartment not tested or result not reported. Serial Sampling: longitudinal specimen collection at ≥2 timepoints. Shedding Duration: longest documented RNA-positive timepoint per compartment within each study. GCF = gingival cervical fluid.

## Data Availability

All data generated or analyzed during this research are included in the manuscript. Further inquiries can be directed to the corresponding author.

## Supplementary Materials

The following supporting information can be downloaded at: https://www.mdpi.com/article/10.3390/v18070699/s1, Table S1: Full Search Strategies by Database; Table S2: PRISMA checklist 2020; Table S3: Geographical setting and transmission timing data of included studies.

## Abbreviations

The following abbreviations are used in this manuscript:

BP	Blood/plasma
CT	Contact tracing
GCF	Gingival cervical fluid
HCPS	Hantavirus cardiopulmonary syndrome
HCW	Healthcare worker exposure
HFRS	Hemorrhagic fever with renal syndrome
HH	Household/family contact
HI	Hospital isolation
IC	Infection control
MM	Multiple measures
MS	Multiple specimen type;
P2P	Person-to-person transmission;
P2Pd	Person-to-person documented
PH	Public health
PPE	Personal protective equipment
SAR	Secondary attack rate
SWiM	Synthesis Without Meta-analysis
Z	Zoonotic transmission/rodent exposure.
