# Supplementary material for: Person-to-Person Transmission of Andes Virus (ANDV): A Systematic Review of Transmission Dynamics, Viral Shedding, and Public Health Implications
Source: Viruses. 2026 Jun 25;18(7):699. doi: 10.3390/v18070699 (PMC13431334; doi:10.3390/v18070699)
Supplement: Supplementary file 1 [file viruses-18-00699-s001.zip › viruses-4376943-supplementary.pdf]

Supplementary Table S1. Full Search Strategies by Database.

The search was conducted from database inception to 14 May 2026 across three bibliographic databases. For PubMed/MEDLINE, the strategy combined Medical Subject Headings (MeSH) terms with free-text keywords in title and abstract ([tiab]) using Boolean operators. Database-specific syntax was applied for Scopus (TITLE-ABS-KEY) and Web of Science Core Collection (Topic field, TS). No date, language, or study design restrictions were applied at the database level.

Database	Search Date	Field Tag	Full Search String	Records Retrieved
PubMed / MEDLINE	14 May 2026	MeSH + [tiab]	(<br>"Andes virus"[tiab] OR "Andes hantavirus"[tiab]<br>OR "Andes orthohantavirus"[tiab]<br>OR "Orthohantavirus andesense"[tiab]<br>OR "Andes orthohantavirus"[Supplementary Concept]<br>OR ANDV[tiab]<br>OR (<br>(<br>"Hantavirus Pulmonary Syndrome"[Mesh]<br>OR "Hantavirus Infections"[Mesh]<br>OR "hantavirus pulmonary syndrome"[tiab]<br>OR "hantavirus cardiopulmonary syndrome"[tiab]<br>OR HCPS[tiab] OR hantavirus[tiab] OR hantaviruses[tiab]<br>)<br>AND<br>(<br>Andes[tiab] OR Argentina[tiab] OR Chile[tiab]<br>OR Patagonia[tiab] OR "South America"[tiab]<br>OR "Oligoryzomys longicaudatus"[tiab]<br>OR Oligoryzomys[tiab] OR colilargo[tiab]<br>)<br>)<br>)<br>AND<br>(<br>transmission[tiab] OR transmissibility[tiab]<br>OR "person-to-person"[tiab] OR "person to person"[tiab]<br>OR "human-to-human"[tiab] OR "human to human"[tiab]<br>OR interhuman[tiab] OR "inter-human"[tiab]<br>OR contagious*[tiab] OR infectiousness[tiab] OR infectivity[tiab]<br>OR "secondary transmission"[tiab] OR "secondary case"[tiab]<br>OR "secondary cases"[tiab] OR "secondary infection"[tiab]	486

Database	Search Date	Field Tag	Full Search String	Records Retrieved
			OR "secondary infections"[tiab]<br>OR "transmission chain"[tiab] OR "transmission chains"[tiab]<br>OR "chain of transmission"[tiab] OR "chains of transmission"[tiab]<br>OR "household transmission"[tiab] OR "household contact"[tiab]<br>OR "household contacts"[tiab] OR "close contact"[tiab]<br>OR "close contacts"[tiab] OR "nosocomial transmission"[tiab]<br>OR "healthcare-associated"[tiab] OR "health care-associated"[tiab]<br>OR "healthcare worker"[tiab] OR "healthcare workers"[tiab]<br>OR "health care worker"[tiab] OR "health care workers"[tiab]<br>OR outbreak*[tiab] OR cluster*[tiab] OR epidemic*[tiab]<br>OR "sporadic case"[tiab] OR "sporadic cases"[tiab]<br>OR "index case"[tiab] OR "primary case"[tiab]<br>OR superspread*[tiab] OR "super-spread"[tiab]<br>OR "super-spreading"[tiab] OR "super spreading"[tiab]<br>OR "contact tracing"[tiab] OR "Contact Tracing"[Mesh]<br>OR quarantine[tiab] OR quarantin*[tiab] OR "Quarantine"[Mesh]<br>OR isolation[tiab] OR isolat*[tiab] OR "Patient Isolation"[Mesh]<br>OR "infection control"[tiab] OR "Infection Control"[Mesh]<br>OR "public health"[tiab] OR "Public Health"[Mesh]<br>OR "risk assessment"[tiab] OR "Risk Assessment"[Mesh]<br>OR "case definition"[tiab] OR "case definitions"[tiab]<br>OR "public health measure"[tiab] OR "public health measures"[tiab]<br>OR "control measure"[tiab] OR "control measures"[tiab]<br>OR "viral load"[tiab] OR "Viral Load"[Mesh]<br>OR viremia[tiab] OR viraemia[tiab] OR "Viremia"[Mesh]<br>OR shedding[tiab] OR "viral shedding"[tiab]<br>OR saliva[tiab] OR "Saliva"[Mesh]<br>OR "oral fluid"[tiab] OR "oral fluids"[tiab]<br>OR "oral swab"[tiab] OR "oral swabs"[tiab]<br>OR "nasopharyngeal swab"[tiab] OR "nasopharyngeal swabs"[tiab]<br>OR "oropharyngeal swab"[tiab] OR "oropharyngeal swabs"[tiab]<br>OR "respiratory secretion"[tiab] OR "respiratory secretions"[tiab]<br>OR "gingival crevicular fluid"[tiab] OR "buffy coat"[tiab]<br>OR PCR[tiab] OR "RT-PCR"[tiab]<br>OR "reverse transcription polymerase chain reaction"[tiab]<br>OR RNA[tiab]<br>OR asymptomatic[tiab] OR "Asymptomatic Infections"[Mesh]<br>OR presymptomatic[tiab] OR "pre-symptomatic"[tiab]	

Database	Search Date	Field Tag	Full Search String	Records Retrieved
			OR paucisymptomatic[tiab] OR "pauci-symptomatic"[tiab]<br>OR incubation[tiab] OR "incubation period"[tiab]<br>OR "Incubation Period"[Mesh]<br>OR "serial interval"[tiab] OR "generation interval"[tiab]<br>OR reservoir*[tiab] OR "natural reservoir"[tiab]<br>OR "animal reservoir"[tiab]<br>OR "Oligoryzomys longicaudatus"[tiab]<br>OR Oligoryzomys[tiab] OR colilargo[tiab]<br>OR ecology[tiab] OR ecological[tiab]<br>OR "geographic distribution"[tiab]<br>OR "geographical distribution"[tiab]<br>OR "risk map"[tiab] OR "risk maps"[tiab] OR "risk mapping"[tiab]<br>OR passenger*[tiab] OR ship[tiab] OR ships[tiab]<br>OR cruise[tiab] OR cruises[tiab] OR vessel[tiab] OR vessels[tiab]<br>)	
Scopus	14 May 2026	TITLE-ABS-KEY	TITLE-ABS-KEY(<br>(<br>"Andes virus" OR "Andes hantavirus"<br>OR "Andes orthohantavirus"<br>OR "Orthohantavirus andesense" OR ANDV<br>OR (<br>(<br>"hantavirus pulmonary syndrome"<br>OR "hantavirus cardiopulmonary syndrome"<br>OR HCPS OR hantavirus OR hantaviruses<br>)<br>AND<br>(<br>Andes OR Argentina OR Chile OR Patagonia<br>OR "South America" OR "Oligoryzomys longicaudatus"<br>OR Oligoryzomys OR colilargo<br>)<br>)<br>)<br>AND<br>(<br>transmission OR transmissibility	613

Database	Search Date	Field Tag	Full Search String	Records Retrieved
			OR "person-to-person" OR "person to person"<br>OR "human-to-human" OR "human to human"<br>OR interhuman OR "inter-human"<br>OR contagious* OR infectiousness OR infectivity<br>OR "secondary transmission" OR "secondary case"<br>OR "secondary cases" OR "secondary infection"<br>OR "secondary infections"<br>OR "transmission chain" OR "transmission chains"<br>OR "chain of transmission" OR "chains of transmission"<br>OR "household transmission" OR "household contact"<br>OR "household contacts" OR "close contact"<br>OR "close contacts" OR "nosocomial transmission"<br>OR "healthcare-associated" OR "health care-associated"<br>OR "healthcare worker" OR "healthcare workers"<br>OR "health care worker" OR "health care workers"<br>OR outbreak* OR cluster* OR epidemic*<br>OR "sporadic case" OR "sporadic cases"<br>OR "index case" OR "primary case"<br>OR superspread* OR "super-spread"<br>OR "super-spreading" OR "super spreading"<br>OR "contact tracing" OR quarantine OR quarantin*<br>OR isolation OR isolat* OR "infection control"<br>OR "public health" OR "risk assessment"<br>OR "case definition" OR "case definitions"<br>OR "public health measure" OR "public health measures"<br>OR "control measure" OR "control measures"<br>OR "viral load" OR viremia OR viraemia<br>OR shedding OR "viral shedding" OR saliva<br>OR "oral fluid" OR "oral fluids"<br>OR "oral swab" OR "oral swabs"<br>OR "nasopharyngeal swab" OR "nasopharyngeal swabs"<br>OR "oropharyngeal swab" OR "oropharyngeal swabs"<br>OR "respiratory secretion" OR "respiratory secretions"<br>OR "gingival crevicular fluid" OR "buffy coat"<br>OR PCR OR "RT-PCR"<br>OR "reverse transcription polymerase chain reaction"<br>OR RNA OR asymptomatic OR presymptomatic<br>OR "pre-symptomatic" OR paucisymptomatic	

Database	Search Date	Field Tag	Full Search String	Records Retrieved
			OR "pauci-symptomatic" OR incubation<br>OR "incubation period" OR "serial interval"<br>OR "generation interval" OR reservoir*<br>OR "natural reservoir" OR "animal reservoir"<br>OR "Oligoryzomys longicaudatus"<br>OR Oligoryzomys OR colilargo<br>OR ecology OR ecological<br>OR "geographic distribution"<br>OR "geographical distribution"<br>OR "risk map" OR "risk maps" OR "risk mapping"<br>OR passenger* OR ship OR ships<br>OR cruise OR cruises OR vessel OR vessels<br>)<br>)	
Web of Science (Core Collection)	14 May 2026	TS (Topic)	TS=(<br>(<br>"Andes virus" OR "Andes hantavirus"<br>OR "Andes orthohantavirus"<br>OR "Orthohantavirus andesense" OR ANDV<br>OR (<br>(<br>"hantavirus pulmonary syndrome"<br>OR "hantavirus cardiopulmonary syndrome"<br>OR HCPS OR hantavirus OR hantaviruses<br>)<br>AND<br>(<br>Andes OR Argentina OR Chile OR Patagonia<br>OR "South America" OR "Oligoryzomys longicaudatus"<br>OR Oligoryzomys OR colilargo<br>)<br>)<br>)<br>AND<br>(<br>transmission OR transmissibility<br>OR "person-to-person" OR "person to person"	619

Database	Search Date	Field Tag	Full Search String	Records Retrieved
			OR "human-to-human" OR "human to human"<br>OR interhuman OR "inter-human"<br>OR contagious* OR infectiousness OR infectivity<br>OR "secondary transmission" OR "secondary case"<br>OR "secondary cases" OR "secondary infection"<br>OR "secondary infections"<br>OR "transmission chain" OR "transmission chains"<br>OR "chain of transmission" OR "chains of transmission"<br>OR "household transmission" OR "household contact"<br>OR "household contacts" OR "close contact"<br>OR "close contacts" OR "nosocomial transmission"<br>OR "healthcare-associated" OR "health care-associated"<br>OR "healthcare worker" OR "healthcare workers"<br>OR "health care worker" OR "health care workers"<br>OR outbreak* OR cluster* OR epidemic*<br>OR "sporadic case" OR "sporadic cases"<br>OR "index case" OR "primary case"<br>OR superspread* OR "super-spread"<br>OR "super-spreading" OR "super spreading"<br>OR "contact tracing" OR quarantine OR quarantin*<br>OR isolation OR isolat* OR "infection control"<br>OR "public health" OR "risk assessment"<br>OR "case definition" OR "case definitions"<br>OR "public health measure" OR "public health measures"<br>OR "control measure" OR "control measures"<br>OR "viral load" OR viremia OR viraemia<br>OR shedding OR "viral shedding" OR saliva<br>OR "oral fluid" OR "oral fluids"<br>OR "oral swab" OR "oral swabs"<br>OR "nasopharyngeal swab" OR "nasopharyngeal swabs"<br>OR "oropharyngeal swab" OR "oropharyngeal swabs"<br>OR "respiratory secretion" OR "respiratory secretions"<br>OR "gingival crevicular fluid" OR "buffy coat"<br>OR PCR OR "RT-PCR"<br>OR "reverse transcription polymerase chain reaction"<br>OR RNA OR asymptomatic OR presymptomatic<br>OR "pre-symptomatic" OR paucisymptomatic<br>OR "pauci-symptomatic" OR incubation	

Database	Search Date	Field Tag	Full Search String	Records Retrieved
			OR "incubation period" OR "serial interval"<br>OR "generation interval" OR reservoir*<br>OR "natural reservoir" OR "animal reservoir"<br>OR "Oligoryzomys longicaudatus"<br>OR Oligoryzomys OR colilargo<br>OR ecology OR ecological<br>OR "geographic distribution"<br>OR "geographical distribution"<br>OR "risk map" OR "risk maps" OR "risk mapping"<br>OR passenger* OR ship OR ships<br>OR cruise OR cruises OR vessel OR vessels<br>)<br>)	
			**Total records retrieved (before deduplication)**	**1,718**

Supplementary Table 2: PRISMA checklist 2020

Section and Topic	Item #	Checklist item	Location where item is reported
**TITLE**			
Title	1	Identify the report as a systematic review.	Pag 1
**ABSTRACT**			
Abstract	2	See the PRISMA 2020 for Abstracts checklist.	Pag 1 line 14
**INTRODUCTION**			
Rationale	3	Describe the rationale for the review in the context of existing knowledge.	Pag 1 line 37
Objectives	4	Provide an explicit statement of the objective(s) or question(s) the review addresses.	Pag 3 Line 99
**METHODS**			
Eligibility criteria	5	Specify the inclusion and exclusion criteria for the review and how studies were grouped for the syntheses.	Pag 3 lines120
Information sources	6	Specify all databases, registers, websites, organisations, reference lists and other sources searched or consulted to identify studies. Specify the date when each source was last searched or consulted.	Pag 4 line 164
Search strategy	7	Present the full search strategies for all databases, registers and websites, including any filters and limits used.	Supplementary table 1
Selection process	8	Specify the methods used to decide whether a study met the inclusion criteria of the review, including how many reviewers screened each record and each report retrieved, whether they worked independently, and if applicable, details of automation tools used in the process.	Pag 4 Line 179
Data collection process	9	Specify the methods used to collect data from reports, including how many reviewers collected data from each report, whether they worked independently, any processes for obtaining or confirming data from study investigators, and if applicable, details of automation tools used in the process.	Pag 5 Line 190
Data items	10a	List and define all outcomes for which data were sought. Specify whether all results that were compatible with each outcome domain in each study were sought (e.g. for all measures, time points, analyses), and if not, the methods used to decide which results to collect.	Pag 5
	10b	List and define all other variables for which data were sought (e.g. participant and intervention characteristics, funding sources). Describe any assumptions made about any missing or unclear information.	Pag 5
Study risk of bias assessment	11	Specify the methods used to assess risk of bias in the included studies, including details of the tool(s) used, how many reviewers assessed each study and whether they worked independently, and if applicable, details of automation tools used in the process.	Pag 6 line 244
Effect measures	12	Specify for each outcome the effect measure(s) (e.g. risk ratio, mean difference) used in the synthesis or presentation of results.	Not Applicable
Synthesis methods	13a	Describe the processes used to decide which studies were eligible for each synthesis (e.g. tabulating the study intervention characteristics and comparing against the planned groups for each synthesis (item #5)).	Pag 4 line 179
	13b	Describe any methods required to prepare the data for presentation or synthesis, such as handling of missing summary statistics, or data conversions.	Pag 5 line 190
	13c	Describe any methods used to tabulate or visually display results of individual studies and syntheses.	Pag 5 line 224
	13d	Describe any methods used to synthesize results and provide a rationale for the choice(s). If meta-analysis was performed, describe the model(s), method(s) to identify the presence and extent of statistical heterogeneity, and software package(s) used.	Pag 5 line 224

Section and Topic	Item #	Checklist item	Location where item is reported
	13e	Describe any methods used to explore possible causes of heterogeneity among study results (e.g. subgroup analysis, meta-regression).	Pag 5 line 224
	13f	Describe any sensitivity analyses conducted to assess robustness of the synthesized results.	Not Applicable
Reporting bias assessment	14	Describe any methods used to assess risk of bias due to missing results in a synthesis (arising from reporting biases).	Pag 6
Certainty assessment	15	Describe any methods used to assess certainty (or confidence) in the body of evidence for an outcome.	Not Applicable
**RESULTS**			
Study selection	16a	Describe the results of the search and selection process, from the number of records identified in the search to the number of studies included in the review, ideally using a flow diagram.	Pag 6 line 259
	16b	Cite studies that might appear to meet the inclusion criteria, but which were excluded, and explain why they were excluded.	Pag 6 line 259
Study characteristics	17	Cite each included study and present its characteristics.	Tables 1-2
Risk of bias in studies	18	Present assessments of risk of bias for each included study.	Figure 5
Results of individual studies	19	For all outcomes, present, for each study: (a) summary statistics for each group (where appropriate) and (b) an effect estimate and its precision (e.g. confidence/credible interval), ideally using structured tables or plots.	Table 1-3
Results of syntheses	20a	For each synthesis, briefly summarise the characteristics and risk of bias among contributing studies.	Pag 21
	20b	Present results of all statistical syntheses conducted. If meta-analysis was done, present for each the summary estimate and its precision (e.g. confidence/credible interval) and measures of statistical heterogeneity. If comparing groups, describe the direction of the effect.	Pag 21
	20c	Present results of all investigations of possible causes of heterogeneity among study results.	Pag 7
	20d	Present results of all sensitivity analyses conducted to assess the robustness of the synthesized results.	Not Applicable
Reporting biases	21	Present assessments of risk of bias due to missing results (arising from reporting biases) for each synthesis assessed.	Not Applicable
Certainty of evidence	22	Present assessments of certainty (or confidence) in the body of evidence for each outcome assessed.	Not Applicable
**DISCUSSION**			
Discussion	23a	Provide a general interpretation of the results in the context of other evidence.	Pag 23 line 548
	23b	Discuss any limitations of the evidence included in the review.	Pag 25 line 653
	23c	Discuss any limitations of the review processes used.	Pag 25 line 653
	23d	Discuss implications of the results for practice, policy, and future research.	Pag 24 line 595
**OTHER INFORMATION**			

Section and Topic	Item #	Checklist item	Location where item is reported
Registration and protocol	24a	Provide registration information for the review, including register name and registration number, or state that the review was not registered.	Pag 3 line 109
	24b	Indicate where the review protocol can be accessed, or state that a protocol was not prepared.	Pag 3 line 109
	24c	Describe and explain any amendments to information provided at registration or in the protocol.	No amendments from the protocol
Support	25	Describe sources of financial or non-financial support for the review, and the role of the funders or sponsors in the review.	Pag 26 line 699
Competing interests	26	Declare any competing interests of review authors.	Pag 26 line 705
Availability of data, code and other materials	27	Report which of the following are publicly available and where they can be found: template data collection forms; data extracted from included studies; data used for all analyses; analytic code; any other materials used in the review.	They can be found in the protocol registered in prospero

Supplementary Table 3. Geographical setting and transmission timing data of included studies.

First Author (year)	Country Region Detail	Incubation Period (days)	Serial Interval Value (days)
Alonso D.O. (2019) [22]	Región de los Ríos	40	NR
Alonso D.O. (2020) [10]	Alemanía, Department of Guachipas, Salta province, northwestern Argentina	P1 to P2: 15 ; P2 to P3: 19–20	P1→P2: 15 days; P2→P3: 22 days
Alonso D.O. (2024) [23]	Imported ANDV infection after travel/trekking from Ecuador to Chile, with illness after return to Switzerland	NR	20
Barrera A. (2025) [14]	Argentina; Central/East, North/West, South/West regions	NR	NR
Bellomo C.M. (2015) [25]	Santiago, Chile	NR	NR
Bellomo C.M. (2025) [24]	Epuyén, Chubut Province	NR	NR
Busch M. (2004) [26]	El Bolsón, Río Negro Province	NR	NR
Cantoni G. (1997) [27]	Southern Chile	NR	3, 9, 20, 27
Castillo C. (2000) [28]	Central and southern Chile	NR	NR

Castillo C. (2001) [29]	Salta and Jujuy provinces, northern Argentina	NR	NR
Della Valle M.G. (2002) [30]	Delaware, United States (travel history to Andes region of Argentina and Chile)	15	NR
Ferrés M. (2007) [6]	12 health centers from 9 Chilean cities	14-30	19.5
Ferrés M. (2020) [31]	Southern Chile (Osorno and Puerto Montt, Los Lagos Region / X Región)	17–26	6 after delivery(mother synthom); 26 days after maternal symptom onset-32 after born (child)
Ferrés M. (2024) [12]	Buenos Aires Province (127 departments analised)	NR	NR
Godoy P. (2009) [32]	Temuco, IX Region, Chile	NR	NR
Iglesias A.A. (2022) [33]	Argentina, Bolivia, Chile, Paraguay, Uruguay	9–40 (median 26)	NR
Kofman A. (2018) [34]	Río Negro Province, Argentina; El Bolsón and San Carlos de Bariloche	42	NR

Lázaro M.E. (2007) [35]	Parral, Chile	NR	19-40
López R. (2019) [36]	Central-East Argentina, especially Buenos Aires province	NR	NR
Lopez R. (2021) [37]	El Bolsón, Bariloche, Esquel, Jacobacci, Futalaufquén National Park, southern Argentina / Patagonia	NR	NR
Martinez V.P. (2005) [9]	Western strip of Neuquen, Rio Negro, Chubut, and Santa Cruz provinces	15 for C4-b,<br>24 to 26 for C1-s, 18 to 22 for C4-c	NR
Martinez V.P. (2010) [38]	Chile	11	NR
Martinez V.P. (2020) [7]	Provinces of Buenos Aires and Neuquén and ANDV–endemic regions,	9–40	23 ± 7
Martinez-Valdebenito C. (2014) [17]	Bermejo,Bolivia	30 (median)	NR
Muñoz-Zanzi C. (2015) [39]	Temuco, Chile	NR	NR
Padula P. (2002) [40]	Coyhaique, Cisne Medio, Villa Amengua	NR	NR
Padula P.J. (2001) [3]	10 collaborative	19.2 (mean)	16, 18, 19, 21, 25, 29

	research hospitals in Chile		
Pizarro E. (2020) [13]	Santiago, Chile	NR	NR
Riquelme R. (2003) [41]	4 region: Northwest, Northeast, Central, and Patagonia	NR	NR
Toro J. (1998) [42]	Buenos Aires, Argentina	NR	C1: P1 -> P2 = 18 ; (C2): P1 -> P2 = 21 ; (C3): P1 -> P2 = 23
Vial P.A. (2006) [11]	12 communities	NR	NR
Wells R. (1997) [43]	Región de los Ríos	NR	21.8
Zust R. (2023) [15]	5 region: NOA, NEA, Central, Cuyo, Patagonia	NR	NR
